# Antioxidants and Retinal Diseases

**DOI:** 10.3390/antiox8120604

**Published:** 2019-11-29

**Authors:** María Miranda, Francisco Javier Romero

**Affiliations:** 1 Departamento Ciencias Biomédicas, Facultad de Ciencias de la Salud, Universidad Cardenal Herrera-CEU, CEU Universities, 46315 Valencia, Spain; 2 Facultad de Ciencias de la Salud, Universidad Europea de Valencia, 46010 Valencia, Spain; jromerogomez@gmail.com; 3 Hospital General de Requena, Generalitat Valenciana, 46340 Valencia, Spain

The retina is a thin membrane derived from the neuroectoderm, it is the physical morphological substrate in which the transformation of light energy into electrical impulses, that later will be led to the cerebral cortex, is performed. Due to its prosencephalic embryological origin, the retina is normally considered a specially differentiated part of the brain. It is a very complex tissue, formed by multiple cell layers and by several types of neuronal cells (ganglion, bipolar, horizontal, amacrine, and photoreceptor cells), microglia (macrophages), macroglia (Müller cells, astrocytes), and vascular cells (endothelium and pericytes). Under physiological conditions, the retina is characterized by a high oxygen consumption rate, intense exposition to pro-oxidizing agents (i.e., light) and a high content of polyunsaturated fatty acids (especially in the photoreceptor membranes). Therefore, retina is especially susceptible to oxidative stress [1,2,3].

Oxidative stress is defined as the imbalance between the generation and elimination of reactive oxygen species (ROS) and it results from either excessive ROS or an impaired antioxidant system. To cope with ROS increase, the retina has evolved different antioxidants defenses such as vitamin E, ascorbate, catalase, glutathione (GSH), glutathione-peroxidase, and glutathione-transferases [3]. However, different pathologies or increasing age may reduce this antioxidant capacity. ROS increase may cause damage to deoxyribonucleic acid (DNA), proteins, and lipids and have been also related to cell death via apoptosis and to proinflammatory and proangiogenic situations [4].

Oxidative stress has been related with a great number of retinal pathologies including diabetic retinopathy, age-related macular degeneration (AMD), glaucoma, and retinal dystrophies [5,6,7,8,9]. It has been suggested that although all these diseases have different etiologies and pathogeneses, they share similar functional and morphological responses [10]. In this sense, one of the functional changes observed in these diseases is an oxidative stress increase that may be linked directly to alterations (such as an increase in ROS associated with glucose metabolism) or may increase the retinal apoptosis observed in other pathologies (i.e., glaucoma or retinitis pigmentosa) [10].

All possible retinal malfunctions and diseases will severely affect the quality of life of patients because the retina allows the phenomenon of vision that is essential for the development of everyday life, and damage to the retina, as a part of the visual system, can lead to blindness.

The hypothetical role of oxidative stress in the development of retinal diseases has given rise to research on the use of antioxidants as preventive and therapeutic agents in retinal therapy. Although the use of antioxidants seems promising to improve results in eye disorders, more research, mainly related to bioavailability, distribution, and interactions between antioxidant molecules, is necessary to standardize the indications for its use, composition, and dose. 

This Special Issue consists of a set of articles which highlight different aspects of antioxidants and retinal tissue in health and disease. These papers illustrate the role of substances or molecules such as saffron, quercetin, ethylmethylhydroxypyridine malate, coenzyme Q10, abscisic acid, progesterone, lipoic acid, and progesterone, as neuroprotective agents in retinal degeneration situations.

Di Marco et al. [11] suggest that saffron may be a therapeutic coadjuvant in age-related macular degeneration (AMD) as it helps in maintaining retinal morphology, probably because of the antioxidant properties of some of its chemical components such as crocins, but also because it reduces metalloproteinase activation. The same authors suggest the beneficial actions of saffron in an experimental model of light-induced retinal degeneration [11]. Another substance suggested to be useful in ischemic retinopathies is the phytohormone, abscisic acid. Its role in modulating glucose metabolism, inflammation, and oxidative stress is reviewed by Baliño et al. [12]. Another drug that has shown to have protective effects in retinal ischemia is the new 3-hydroxypyridine derivative isomer of ethylmethylhydroxypyridine malate [13].

Different animal models can be used to study retinitis pigmentosa (RP), a disease with no cure. Among the most used models are the light-induced retinal degeneration model and the rd1 and rd10. Koyama et al. [14] study the cytoprotective effect of quercetin via activator protein in Sprague Dawley rats with light-induced retinal damage. Hernández-Rabaza et al. [15] review how different drugs such as progesterone, lipoic acid, and sulforaphane are not able to correct the genetic alteration observed in different RP animal models but are able to decrease photoreceptor cell death rate. These results may be particularly important because they imply that patient blindness may be delayed several years. Another well-known antioxidant is coenzyme Q10; it has been shown to scavenge oxygen free and regenerate vitamin E. Beharry el al. [16] suggest that the administration of coenzyme Q10 reduces new retinal vascularization and at the same time improves astrocytes’ morphology in a rat model of oxygen-induced retinopathy.

Nitric oxide (NO) is a gas molecule with diverse physiological and cellular functions in the retina, however, products generated from NO (i.e., dinitrogen trioxide (N2O3) and peroxynitrite) have great oxidative damaging effects. Cantó et al. [17] review the role of NO in several ocular diseases, such as diabetic retinopathy, retinitis pigmentosa, glaucoma, and AMD. The authors report that new therapies targeting nitrosative stress may be effective in retinal pathologies, nevertheless this has to be studied carefully because an excess of NO may also lead to undesired complications.

In conclusion, the studies regarding the use of antioxidants in retinal diseases are complex but support antioxidant supplements as therapeutic aids.
